# West Nile Virus Infection of Birds, Mexico

**DOI:** 10.3201/eid1712.110294

**Published:** 2011-12

**Authors:** Sergio Guerrero-Sánchez, Sandra Cuevas-Romero, Nicole M. Nemeth, María Teresa Jesús Trujillo-Olivera, Gabriella Worwa, Alan Dupuis, Aaron C. Brault, Laura D. Kramer, Nicholas Komar, José Guillermo Estrada-Franco

**Affiliations:** Zoológico Miguel Alvarez del Toro, Tuxtla Gutiérrez, Mexico (S. Guerrero-Sánchez);; Instituto Nacional de Investigaciones Forestales, Mexico City, Mexico (S. Cuevas-Romero);; Centers for Disease Control and Prevention, Fort Collins, Colorado, USA (N.M. Nemeth, A.C. Brault, N. Komar);; Universidad Autónoma de Chiapas, Tuxtla Gutiérrez (M.T. Jesus Trujillo-Olivera);; University of California, Davis, California, USA (G. Worwa);; New York State Department of Health, Slingerlands, New York, USA (A Dupuis, L.D. Kramer);; University of Texas Medical Branch, Galveston, Texas, USA (J.G. Estrada-Franco)

**Keywords:** Mexico, West Nile virus, experimental infection, passerine bird, viruses

## Abstract

West Nile virus (WNV) has caused disease in humans, equids, and birds at lower frequency in Mexico than in the United States. We hypothesized that the seemingly reduced virulence in Mexico was caused by attenuation of the Tabasco strain from southeastern Mexico, resulting in lower viremia than that caused by the Tecate strain from the more northern location of Baja California. During 2006–2008, we tested this hypothesis in candidate avian amplifying hosts: domestic chickens, rock pigeons, house sparrows, great-tailed grackles, and clay-colored thrushes. Only great-tailed grackles and house sparrows were competent amplifying hosts for both strains, and deaths occurred in each species. Tecate strain viremia levels were higher for thrushes. Both strains produced low-level viremia in pigeons and chickens. Our results suggest that certain avian hosts within Mexico are competent for efficient amplification of both northern and southern WNV strains and that both strains likely contribute to bird deaths.

## Abstract 2

Birds of 2 of 3 passerine species died after experimental infection with 2 strains from Mexico.

In Mexico, West Nile virus (WNV; family *Flaviviridae*, genus *Flavivirus*) was first isolated in 2003 from a common raven (*Corvus corax*) carcass in Tabasco (southeast Mexico) (*1*). According to findings of WNV-neutralizing antibodies in horses from the coastal states of eastern Mexico and in resident birds in the Yucatan Peninsula, the virus had spread to Mexico at least 1 year earlier (*1**–**3*). In the United States and Canada, morbidity and mortality rates for WNV infection are high among humans, horses, and birds; but in Mexico and other regions of Latin America, the health effects of this virus remain unknown (*4*). Low numbers of cases in humans, equids, and birds in Mexico have been reported, primarily from the northern border with the United States, where isolated WNV strains (e.g., Tecate) were genetically related to the North American 2002 strain circulating in the southwestern United States (*5*).

The paucity of reported WNV cases in Mexico might be the result of multiple factors involved in local virus ecology. The interactions of amplifying hosts, vectors, and virus strains in Mexico, combined with external factors such as climate, habitat, and circulation of interfering flaviviruses, may result in relatively low levels of transmission and disease. Virus–host interactions in Mexico, including susceptibility and competence of candidate amplifying hosts, remain unknown. Assessment of the response of various avian species to WNV infection could elucidate aspects of the transmission ecology in tropical ecosystems and provide insight for potential surveillance strategies.

To address knowledge gaps regarding transmission and to investigate whether the apparently low prevalence of WNV disease in Mexico could result from reduced virulence of WNV strains from Mexico, during 2006–2008 we experimentally infected birds. We selected birds of several common species as potential WNV-amplifying hosts, including domestic chickens (*Gallus gallus*), rock pigeons (*Columba livia*), house sparrows (*Passer domesticus*), great-tailed grackles (*Quiscalus mexicanus*), and clay-colored thrushes (*Turdus grayi*). We measured viremia, virus shedding, survival rates, and tissue tropism and calculated reservoir competence index values in birds infected with WNV strains from southern Mexico (Tabasco) or northern Mexico (Tecate).

## Materials and Methods

### Experimental Birds

All birds in the study were adults and originated in Mexico. They were either acquired commercially (chickens) or trapped by using mist nets (house sparrows and clay-colored thrushes) or walk-in traps (rock pigeons and great-tailed grackles). The birds were moved to indoor housing, where blood samples were collected and serum was tested for neutralizing antibodies to WNV as determined by plaque-reduction neutralization test (*6*). All birds were cared for in animal holding facilities at the National Institute of Forestry, Agriculture and Livestock, Palo Alto, Mexico City.

### Experimental Inoculation and Sampling Protocol

Low-passage WNV strains originally isolated from tissues harvested from common ravens from southern Mexico (Tabasco; GenBank accession no. AY660002, 7 Vero passages) and northern Mexico (Tecate; GenBank accession no. DQ080060, 2 Vero passages) were used to inoculate birds. Because of the additional passages of the Tabasco strain, we sequenced the viral protein coding region to determine the presence or absence of potential vertebrate virulence determinants, such as the glycosylation motif at positions 154–156 of the envelope (E) protein. Birds seronegative for WNV and St. Louis encephalitis virus (*Flaviviridae*) were subcutaneously inoculated in the pectoral region at concentrations of ≈100,000 Vero PFU/0.1 mL in sterile phosphate-buffered saline. Sample sizes of 4–6 birds were inoculated for each species–virus strain combination, and 1–2 additional birds per group were sham inoculated as negative controls. Blood was collected from all birds (sparrows 0.1 mL; all others 0.2 mL), and oral and cloacal swab samples were collected at ≈24-hour intervals for 6 or 7 days postinoculation (dpi). Coagulated blood was centrifuged to separate serum, which was placed in cryovials. Serum and swab samples were stored at −80°C until tested. A postinfection 0.6-mL blood sample was collected from survivors at 14–28 dpi. All surviving birds were euthanized, and necropsies were performed. The following tissues were collected: heart, kidney, liver, spleen, skin, and brain from all species except pigeons; intestine from thrushes, grackles, and sparrows; and pancreas and lung from grackles and sparrows. Tissues were frozen at −80°C. Some blood samples from chickens and pigeons had been destroyed before viremia could be determined. All animal studies were approved by the US Centers for Disease Control and Prevention Institutional Animal Care and Use Committee 05-26-005-MSA and by the National Institute of Forestry, Agriculture and Livestock Animal Bioethics Committee.

### Laboratory Assays

To determine viral loads in tissue homogenates, swab samples, and serum samples, we used plaque assay for end-point titration in Vero cell culture (*6*). Tissue homogenates were prepared by placing ≈0.5 cm^3^ of each tissue into 2-mL polypropylene tubes containing 1 mL BA-1 medium (medium 199 with Hank balanced salt solution; 0.05 mol/L Tris buffer, pH 7.6; 1% bovine serum albumin, 0.35 g/L of NaHCO_3_, 100 mg/L streptomycin, 100 U/mL penicillin G, 1 μg/mL amphotericin B) supplemented with 20% fetal bovine serum and a 4.5-mm–diameter copper-coated steel bead. Samples were macerated in a mixer mill (Retsch GmbH, Haan, Germany) for 5 min at 25 cycles/s and clarified by centrifugation. Swab samples were soaked in 1 mL BA-1 supplemented with 20% fetal bovine serum and vortexed for 5–10 s. Serum samples were diluted 1:10 in BA-1.

Antibodies were detected in serum samples by using the plaque-reduction neutralization test in Vero cell monolayers prepared in 6-well polystyrene culture plates (*6*). Samples were heat inactivated at 56°C for 30 min and tested for neutralizing antibodies at a 1:10 dilution against WNV strain NY99–4132, originally isolated from the brain of a dead crow in New York, and St. Louis encephalitis virus strain TBH-28, originally isolated from a person in Florida, USA.

### Mathematical and Statistical Analyses

Viremia titers were log transformed for statistical tests. Mean log viremia titers were compared by the Student *t* test, and the Bonferroni adjustment was applied for multiple comparisons. Using the vertebrate reservoir competence index, we analyzed viremia data to determine the potential of each species to infect vector mosquitoes (*7*). Species-specific reservoir competence index values, *C_i_*, were calculated according to the equation *C_i_* = *S* × *I* × *D*, where *S* is susceptibility to infection (0.0–1.0), *I* is mean daily infectiousness (0.0–1.0) with units representing the average proportion of *Culex quinquefasciatus* mosquitoes that are expected to become infectious after feeding on an infectious bird, and *D* is duration, the number of days that viremia remained infectious with titers >10^4.7^ PFU/mL serum for any given bird. Viremia titers below this threshold were considered zero (i.e., not infectious). Infectiousness, *I*, was inferred from viremia measurements according to the formula derived by Kilpatrick et al. (*8*): *I* = 0.1349 × log_10_(viremia) − 0.6235.

Confidence intervals of means were calculated by using the following standard equation:

95% confidence interval = mean ± 1.96 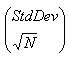


*C_i_* for an arbovirus represents the relative number of vectors that a bird is inherently able to infect during its viremic phase. Overlapping confidence intervals around calculated means indicated lack of significant differences.

## Results

### Viremia and Reservoir Competence Index Values

**Figure 1:**
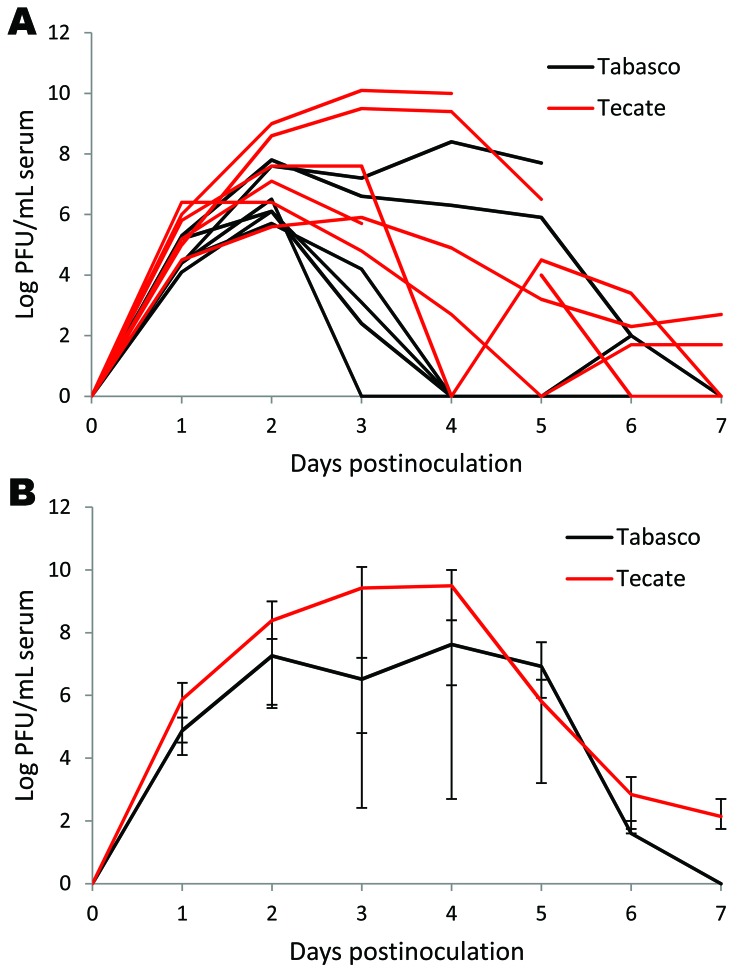
Viremia profile for house sparrows experimentally inoculated with Tabasco or Tecate strains of West Nile virus. Virus titers are plotted on a logarithmic scale. A) Individual birds; B) group means. Error bars represent ranges of observed titers.

**Figure 2:**
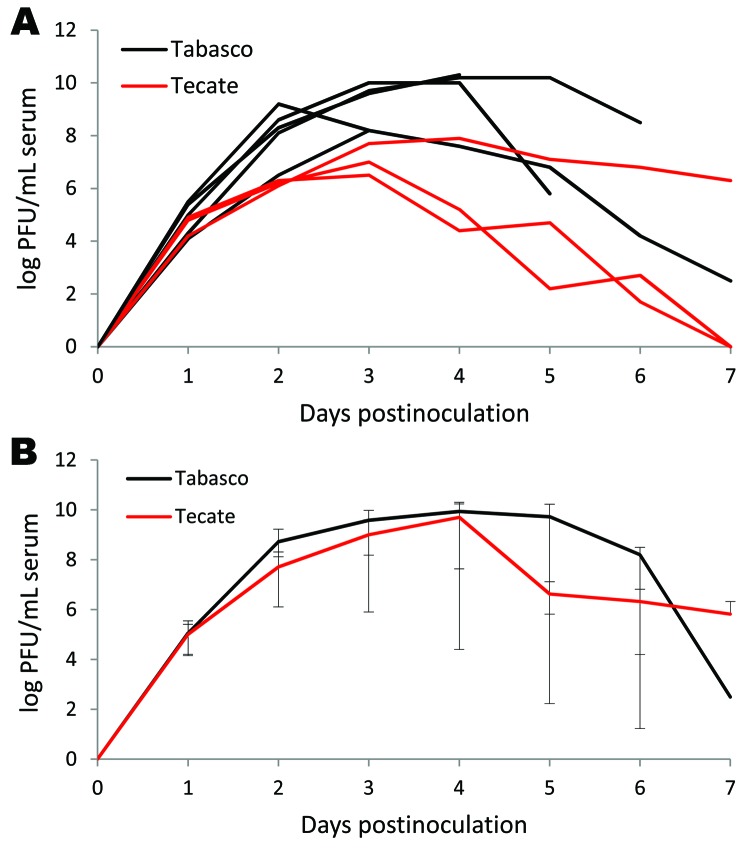
Viremia profile for great-tailed grackles experimentally inoculated with Tabasco or Tecate strains of West Nile virus. Virus titers are plotted on a logarithmic scale. A) Individual birds; B) group means. Error bars represent ranges of observed titers.

**Figure 3:**
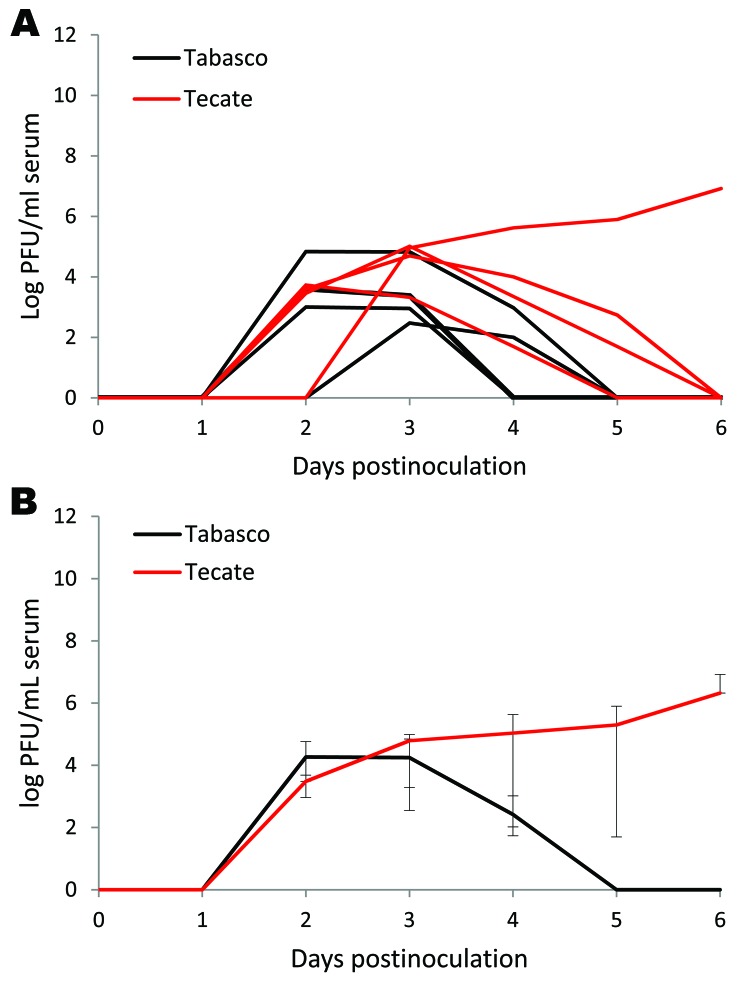
Viremia profile for clay-colored thrush experimentally inoculated with Tabasco or Tecate strains of West Nile virus. Virus titers are plotted on a logarithmic scale. A) Individuals birds; B) group means. Error bars represent ranges of observed titers.

Observed viremia titers for mature chickens and pigeons did not reach infectious levels for mosquitoes, making these birds, at least when adults, incompetent hosts for the 2 strains of WNV from Mexico used in this study. Conversely, the 3 passerine species examined were competent hosts. Log-transformed mean peak viremia titers did not statistically differ between the 2 virus strains for any of the species tested (Table 1). The viremia profiles for each strain did not dramatically differ within a species, except for thrushes, because 1 thrush infected with the Tecate strain maintained a high level of viremia while the others experienced declines (Figure 1, Figure 2, and Figure 3). Among the 3 passerine species, moderately infectious viremia for each of the 2 strains developed in the thrushes, whereas infectiousness for each of the 2 strains was higher for the sparrows and grackles. Also among the 3 passerine species, peak viremia titers differed significantly for the Tabasco strain (p<0.005, α = 0.05 with Bonferroni adjustment for 3 comparisons) but not for the Tecate strain. To evaluate the potential of these passerines to infect vector mosquitoes, we compared reservoir competence index values, which predict the relative number of infectious vectors, i.e., those that will transmit virus after feeding on a bird of each species. An individual sparrow and a thrush were each predicted to generate ≈2- and 20-fold more infectious vectors when infected with Tecate than with Tabasco strain viruses, respectively (Table 1). A grackle infected with the Tabasco strain, however, would generate ≈1.5-fold more infectious mosquitoes. However, none of these quantitative differences were significant. Regardless, a thrush was predicted to infect fewer mosquitoes with either strain than would a grackle or sparrow (confidence intervals around C*_i_* values did not overlap). Thus, among the passerine species tested, the clay-colored thrush seemed to be less of an amplifying host for the 2 WNV strains from Mexico than were house sparrows and great-tailed grackles.

**Table 1:** Viremia parameters and reservoir competence index values for birds from Mexico infected with West Nile virus*

Species	Tecate strain viremia						Tabasco strain viremia				
	No.	Duration (95% CI)†	Infect (95% CI)‡	Mean peak (range)§	Comp (95% CI)¶		No.	Duration (95% CI)†	Infect (95% CI)‡	Mean peak (range)§	Comp (95% CI)¶
House sparrow	6	3.3 (2.8–3.9)	0.34 (0.23–0.44)	9.4 (5.9–10.1)	1.12 (0.43–1.81)		6	2.3 (0.9–3.7)	0.27 (0.20–0.34)	7.7 (5.7–8.4)	0.62 (0.11–1.14)
Great-tailed grackle	4	4.5 (3.8–5.2)	0.28 (0.19–0.38)	9.7 (6.5–10.3)	1.28 (0.49–2.07)		4	4.2 (2.7–5.8)	0.47 (0.36–0.58)	9.8 (8.2–10.2)	2.01 (1.14–2.88)
Clay-colored thrush	4	1.5 (0.3–2.7)	0.12 (0.03–0.21)	6.3 (3.7–6.9)	0.18 (0.00–0.50)		4	0.5 (0.0–1.2)	0.03 (0.03–0.03)	4.3 (2.5–4.8)	0.01 (0.00–0.04)
Domestic chicken	9	0	0	2.9 (0–3.3)#	0.00		11	0	0	3.3 (0–4.1)#	0
Domestic pigeon	11	0	0	2.0 (1.8–2.2)#	0.00		9	0	0	3.4 (1.3–4.0)#	0

*Infect, infectiousness; comp, competence; CI, confidence interval. †No. days that viremia titers were >10^4.7^ PFU/mL serum for any given bird. ‡Average proportion of *Culex quinquefasciatus* mosquitoes that are expected to become infectious vectors after feeding on an infectious bird. §Expressed as log PFU/mL. ¶Species-specific reservoir competence index. Values are calculated as the product of duration, infectiousness, and susceptibility to infection, which was 1.0 for all species in the table. #Mean peak viremia values presented for chickens and pigeons may be underestimated because of intermittent sample collection.

### Shedding

Shedding was evaluated in the same 3 passerine species: great-tailed grackles, house sparrows, and clay-colored thrushes. Thrushes were sampled on 6 and 7 dpi only and were negative (n = 4 for each strain). Most grackles and sparrows orally shed infectious virus of the Tabasco and Tecate strains. Swab sample collection from birds inoculated with the Tabasco strain was not adequate for assessing cloacal shedding, although it was noted for at least 1 grackle and 1 sparrow; cloacal shedding was noted for most birds inoculated with the Tecate strain (Table 2). Oral swab samples generally contained more virus particles than cloacal swab samples, and the Tabasco strain was shed in higher concentrations than the Tecate strain. No shedding was observed in negative control birds.

**Table 2:** Virus shedding by 2 bird species experimentally inoculated with West Nile virus from Mexico*

Species	No.	WNV strain	Oral shedding				Cloacal shedding		
			Peak titer, log PFU/swab†	When detected, dpi	Proportion positive		Peak titer, log PFU/swab†	When detected, dpi	Proportion positive
House sparrow	6	Tecate	5.0	2–7	1.00		1.4	2–3	0.83
	6	Tabasco	7.2	2–6	0.67		4.9	4	NR
Great-tailed grackle	4	Tecate	3.7	2–7	0.75		2.4	3	0.75
	4	Tabasco	6.2	5–6	0.75		4.6	3–7	NR

*dpi, days postinoculation; NR, not reported because of insufficient sample collection. †Peak titers and end point of detection might be underestimated because of intermittent sample collection.

### Illness and Death

During the 7-dpi period, lethargy and fluffed feathers were observed among some grackles and sparrows but not among thrushes, pigeons, or chickens. Birds died within 24 hours after onset of clinical signs. Among sparrows, of the 6 inoculated with the Tabasco strain, 4 (≈67%) died; and of the 6 inoculated with the Tecate strain, 4 (≈67%) also died. The sham-inoculated sparrow showed no signs of illness. Among grackles, of the 4 inoculated with the Tabasco strain, 3 (75%) died; and of the 4 inoculated with the Tabasco strain, 1 (25%) died. Of the 2 sham-inoculated grackles, 1 (50%) died. A high viremia titer developed in 1 thrush at 5 dpi, and the bird was found dead at 8 dpi.

### Tissue Tropism

Viral loads were determined in brain, viscera, and skin from each of 5 sparrows and 4 grackles that died acutely. Sample numbers were too low to detect significant differences in tissue tropisms or viral loads among strains or species. However, high-titered viral loads (>10^7^ PFU/0.5 cm^3^) were found for both WNV strains in brain, heart, spleen, kidney, lung, and skin of sparrows and grackles (Table 3).

**Table 3:** Tissue tropism and viral loads in birds that died after experimental inoculation with WNV, Mexico*

Species†	WNV strain	dpi	Viral load by tissue type, PFU/0.5 cm^3^								
			Brain	Heart	Spleen	Kidney	Lung	Liver	Pancreas	Intestine	Skin
House sparrow	Tecate	4	8.0	7.6	NR	NR	9.3	8.1	4.9	<0.7	7.6
	Tecate	5	8.2	7.7	7.6	7.2	9.2	8.0	5.2	3.1	8.0
	Tabasco	6	2.3	2.9	NR	3.6	4.5	5.7	<0.7	<0.7	4.9
	Tabasco	7	<0.7	<0.7	<0.7	1.3	1.9	<0.7	<0.7	<0.7	<0.7
	Tabasco	7	3.1	7.7	4.8	5.9	5.2	5.2	<0.7	NR	NR
Great-tailed grackle	Tecate	4	7.4	8.2	7.5	8.8	9.3	7.6	6.7	<0.7	8.0
	Tecate	12	3.4	5.1	5.5	5.9	5.2	5.8	6.0	3.0	4.9
	Tabasco	3	NR	8.1	3.8	8.6	8.5	7.9	<0.7	NR	NR
	Tabasco	4	8.1	7.7	6.6	7.9	NR	7.4	4.7	2.7	7.8

*WNV, West Nile virus; dpi, days postinoculation; NR, not reported because of insufficient sample collection. †Deaths occurred in sparrows and grackles but not thrushes and chickens; thrushes and chickens were euthanized on 21 and 7 dpi, respectively, and tissues from these birds were negative for infectious WNV with the exception of 1 chicken-derived skin sample (10^2.0^ PFU/0.5 cm^3^).

### Antibody Responses

All inoculated animals tested for seroconversion at 14–28 dpi had a strong detectable neutralizing antibody response to WNV (100% neutralization by 1:10 serum); there was no noticeable effect of strain used for inoculation (n = 6 sparrows, 3 grackles, 7 thrushes). Sham-inoculated controls showed no evidence of antibodies against WNV or viremia.

### Virus Sequencing

Approximately 11 kb of the Tabasco strain was sequenced, encompassing almost all the genome except the 5′ untranslated region (UTR) and the 20 3′ terminal nucleotides of the 3′ UTR. Comparing the sequence from the consensus TM-171–03 isolate previously published (*5*), the following amino acid differences were identified: prM-T141I, E-P156S, E-V159A, NS3-D401Y, NS4B-V245I, NS5-I898T. The most notable difference was that of a glycosylation motif between E154 and E156. Of the 6 aa differences between the TM-171–03 sequence and the Tabasco virus stock used in this study, 4 of these differences in the stock described herein were synonymous with that of the prototype NY99 strain. The strain used to inoculate birds differed from NY99 at E-V159A (present in all WN02 genotypes) and NS3-D401Y. Several 3′ UTR nucleotide mutations were also noted between the published TM-171–03 and the stock used in this study at c10,772t, g10,828t, and a10,989g. Only the c10,772t transition was unique to this stock; the other mutations were synonymous with the prototype NY99 strain.

## Discussion

The results of inoculation of birds with WNV strains of Mexican origin are difficult to compare with results of published WNV infection studies because of variation in bird species, virus strains, and inoculation method (e.g., needle vs. mosquito bite). House sparrows from Mexico that were needle inoculated with the Tabasco strain were almost as infectious as house sparrows originating in the United States that were needle inoculated with the WNV NY99 strain but 3-fold less infectious than mosquito-inoculated sparrows from the United States (*9*). Responses of adult chickens to needle inoculation with strains from Mexico and the NY99 strain were similar (*10*). Chickens and pigeons consistently failed to become infectious regardless of infection method or virus strain (*11**,**12*). Common grackles (*Quiscalus quiscula*) inoculated by mosquito bite with NY99 have been found to be more competent amplifying hosts than American robins (*Turdus migratorius*) (*12*) in concordance with the findings of our studies that used the 2 strains from Mexico in species of these same genera. However, the American robin was a moderately competent host for NY99, whereas the clay-colored thrush was minimally competent for the Tabasco strain. The 20-fold greater competence of the clay-colored thrush for the Tecate strain was still relatively less than competence for its counterpart from the United States for the NY99 strain and is somewhat contrived because the clay-colored thrush is absent from northwest Mexico where the Tecate strain was isolated. The observed competence of the great-tailed grackle for the Tabasco strain is about as high as or higher than that observed for any species regardless of strain or inoculation method (*8*). This species is likely a major reservoir throughout its range from the central plains of the United States south throughout Mexico and Central America and into Colombia.

Observed differences in quantitative viremia measurements and calculated competence index values between the 2 virus strains from Mexico could not be substantiated by using statistical tests on direct comparisons of like measurements. However, binomial statistics applied across measurement categories did indicate a significant difference. The Tecate strain generally seemed to be more virulent than the Tabasco strain for the avian species examined, except for grackles. One basis for these differing outcomes could be the presence of virulence factors in the viral genome of the Tecate strain that may be absent from the Tabasco strain.

Genetic determinants associated with high titers of viremia in the American crow (*Corvus brachyrhynchos*) include a glycosylation site on the E protein (*5*,*13*) and an amino acid substitution in the NS3 helicase (*14*). The original, unpassaged Tabasco strain was shown to contain at least 2 genotypes, 1 of which was determined to have reduced virulence and lacks the glycosylation site on the E protein (*5*). However, the passaged Tabasco strain we used to inoculate birds from Mexico retained the E protein glycosylation site. Previous data have indicated the association of glycosylation of the E protein for virulence in mice and hatchling chickens (*13*,*15*,*16*). The absence of this reported virulence factor, as well as the presence of additional potential attenuating mutations in the original unpassaged Tabasco strain, could explain the lack of observed bird deaths in Mexico and Latin America, assuming a widespread circulation of this genotype throughout the region. This attenuated phenotype could be explained by selection for lower virulence in migrating birds (*4*). Migrating birds with WNV infections have been shown to maintain their migratory behavior during the viremic phase of infection (*17*). Therefore, viremic migrating birds that survive long-distance (i.e., trans-Gulf) migration to the Tabasco region may have contributed to selection for mutant, low-virulence genomes. However, after serial passage in vertebrate cells, the Tabasco strain used in this study could have reverted to a more virulent phenotype. As evidence of this potential, previous studies have demonstrated that repeated passage of WNV (Kunjin) has resulted in attainment of a glycosylated phenotype after as few as 2 passages in Vero cells (*18*). The 3 additional amino acid differences (prM-T141I, NS4BV245I, or NS5-I898T) in this Tabasco stock that were identical at these positions to the NY99 avian virulent strain could individually or in combination also impart the enhanced avian virulent phenotype observed in these studies.

Both WNV strains from Mexico were pathogenic, leading to death in birds of 2 species (house sparrows and great-tailed grackles), although the stress of captivity and handling may have exacerbated illness among these birds (as indicated by the death of the 1 sham-inoculated grackle). This result signifies that birds in the tropics are probably dying of WNV infection. Therefore, surveillance of bird deaths from WNV may be useful for early warnings of outbreaks in Mexico, as it has been in the United States (*19*), although challenges include lack of public involvement and rapid disappearance of carcasses. The link between surveillance of bird illness and deaths and emerging zoonotic pathogens such as highly pathogenic avian influenza virus (Asian strain subtype H5N1) (*20*) suggests that a large effort should be made to investigate bird deaths.

From our data, thrushes do not appear to be amplifying hosts of the WNV Tabasco strain. However, house sparrows and great-tailed grackles are highly competent hosts and susceptible to infection and some associated deaths, suggesting that high rates of WNV transmission in the American tropics is being overlooked. Alternatively, infection rates are not high among sensitive species such as house sparrows and great-tailed grackles, or these species are fed upon by vectors at lower rates than expected. Blood meal identification studies of *Culex* spp. mosquitoes have demonstrated that these mosquitoes feed on house sparrows and common grackles (*Q. quiscula*) at a frequency lower than expected from the relative abundance of these avian species (*21**,**22*). The same studies report a strong preference of *Culex* spp. mosquitoes for blood meals from American robins. Because clay-colored thrushes were not highly competent hosts for strains of WNV from Mexico, preference of infected *Culex* spp. mosquito vectors for blood meals from this congener could lead to zooprophylaxis. Relatively high viral loads in tissues of birds infected with Tabasco or Tecate WNV strains (i.e., loads greater than or equal to those of birds infected with WNV NY99) (*12*) could result in higher rates of oral transmission to predatory or carrion-eating vertebrates, even if mosquito-borne transmission is less supported in tropical than in temperate regions. For example, feral dogs and cats, raptors, corvids, and other animals may become orally infected by eating WNV-infected birds and carcasses (*23**–**25*). In addition, birds from Mexico that are inoculated with Tabasco and Tecate strains shed infectious WNV from oral and cloacal cavities, as did birds infected with the WNV NY99 strain (*12*). Shedding of WNV could serve as an additional source of non–vector-borne transmission.

This study indicates that WNV is probably contributing to deaths of some species of birds in the tropics, where numerous unique bird populations are often geographically isolated because of islands of fragmented habitats. In contrast, high levels of biodiversity, such as those found in the Neotropics, lend themselves to reduced WNV transmission (*26**,**27*). Urban locations are less biodiverse yet colonized with numerous species of birds competent for amplifying WNV, like house sparrows and, even more so, great-tailed grackles. The lack of peridomestic corvid populations in the Neotropics would seemingly contribute to reduced human risk for WNV infection in the region (*28*). However, there could be an alternative, less susceptible, super spreader, as with the American robin in the United States.

Aside from ecological explanations of reduced WNV transmission in the tropics, human factors may ultimately explain the lack of an obvious public health problem. Three major human factors stand out in this regard: 1) the high incidence of secondary flavivirus infections, mainly caused by dengue virus holoendemicity, which may cause high levels of cross-reactive flavivirus-reactive antibodies; 2) the low investment in surveillance and diagnostic services because of the lack of confirmed human cases of West Nile neurologic disease, and finally, 3) the inability of arbovirus reference laboratories to use serologic methods to diagnose WNV-induced neurologic illness in persons with circulating heterologous antibodies against flavivirus. This last possibility raises concerns that WNV might indeed cause a substantial amount of disease in Mexico, as it does in the United States, but it might be difficult to detect.
